# Impaired vascular function after exposure to diesel exhaust generated at urban transient running conditions

**DOI:** 10.1186/1743-8977-7-19

**Published:** 2010-07-23

**Authors:** Stefan Barath, Nicholas L Mills, Magnus Lundbäck, Håkan Törnqvist, Andrew J Lucking, Jeremy P Langrish, Stefan Söderberg, Christoffer Boman, Roger Westerholm, Jakob Löndahl, Ken Donaldson, Ian S Mudway, Thomas Sandström, David E Newby, Anders Blomberg

**Affiliations:** 1 Department of Public Health and Clinical Medicine, Respiratory Medicine, Umeå University, Umeå, Sweden; 2 Division of Respiratory Medicine and Allergy, Department of Medicine, University Hospital, Umeå, Sweden; 3 Centre for Cardiovascular Science, Edinburgh University, Edinburgh, UK; 4 Department of Public Health and Clinical Medicine, Medicine, Umeå University, Umeå, Sweden; 5 Energy Technology and Thermal Process Chemistry, Umeå University, Umeå, Sweden; 6 Arrhenius Laboratory, Dept of Analytical Chemistry, Stockholm University, Stockholm, Sweden; 7 Division of Nuclear Physics, Department of Physics, Lund University, Lund, Sweden; 8 ELEGI Colt Laboratory, Centre for Inflammation Research, Edinburgh University, UK; 9 King's College London, MRC-HPA Centre for Environment and Health, School of Biomedical and Health Sciences, London, UK

## Abstract

**Background:**

Traffic emissions including diesel engine exhaust are associated with increased respiratory and cardiovascular morbidity and mortality. Controlled human exposure studies have demonstrated impaired vascular function after inhalation of exhaust generated by a diesel engine under idling conditions.

**Objectives:**

To assess the vascular and fibrinolytic effects of exposure to diesel exhaust generated during urban-cycle running conditions that mimic ambient 'real-world' exposures.

**Methods:**

In a randomised double-blind crossover study, eighteen healthy male volunteers were exposed to diesel exhaust (approximately 250 μg/m^3^) or filtered air for one hour during intermittent exercise. Diesel exhaust was generated during the urban part of the standardized European Transient Cycle. Six hours post-exposure, vascular vasomotor and fibrinolytic function was assessed during venous occlusion plethysmography with intra-arterial agonist infusions.

**Measurements and Main Results:**

Forearm blood flow increased in a dose-dependent manner with both endothelial-dependent (acetylcholine and bradykinin) and endothelial-independent (sodium nitroprusside and verapamil) vasodilators. Diesel exhaust exposure attenuated the vasodilatation to acetylcholine (P < 0.001), bradykinin (P < 0.05), sodium nitroprusside (P < 0.05) and verapamil (P < 0.001). In addition, the net release of tissue plasminogen activator during bradykinin infusion was impaired following diesel exhaust exposure (P < 0.05).

**Conclusion:**

Exposure to diesel exhaust generated under transient running conditions, as a relevant model of urban air pollution, impairs vasomotor function and endogenous fibrinolysis in a similar way as exposure to diesel exhaust generated at idling. This indicates that adverse vascular effects of diesel exhaust inhalation occur over different running conditions with varying exhaust composition and concentrations as well as physicochemical particle properties. Importantly, exposure to diesel exhaust under ETC conditions was also associated with a novel finding of impaired of calcium channel-dependent vasomotor function. This implies that certain cardiovascular endpoints seem to be related to general diesel exhaust properties, whereas the novel calcium flux-related effect may be associated with exhaust properties more specific for the ETC condition, for example a higher content of diesel soot particles along with their adsorbed organic compounds.

## Background

Increasing attention has been directed towards the adverse health effects associated with particulate matter (PM) air pollution in terms of both respiratory and cardiovascular morbidity and mortality [1-3]. Changes in air pollution levels over previous decades account for as much as 17% of the change in life expectancy over this period [4]. Several studies have pointed towards traffic-related air pollution as being of particular concern. Living or going to school in proximity to major thoroughfares is associated with reduced lung growth in children and increased risk of asthma, as well as cardiovascular events including increased atherosclerosis and left ventricular hypertrophy in adult populations [5-10].

In a pivotal study, Peters and co-workers demonstrated that patients admitted with myocardial infarction were three times more likely to have been in traffic in the hours prior to the onset of symptoms, suggesting a causal link to the triggering of acute coronary events. The authors identified an association between traffic exposure and the onset of chest pain immediately and six hours prior to the event, indicating that more than one mechanism is likely to be involved [11].

Emissions from motor vehicles, in particular combustion-derived particles from diesel engines, contribute to up to 40% of urban PM and are thought to play a major role in the adverse effects of PM. Experimental exposure studies in healthy and asthmatic individuals have demonstrated oxidative stress, airway inflammation and worsening of asthma, as well as acute cardiovascular events [12-17]. Recent studies have confirmed that diesel exhaust (DE), generated during idling engine conditions, causes endothelial dysfunction, arterial stiffness, decreased fibrinolytic capacity, increased platelet activation and increased *ex-vivo *thrombus formation in the first 24 hours following exposure [12-14,18]. Furthermore, patients with stable coronary heart disease experienced more ST-segment depression with exercise during exposure to diesel exhaust as compared to filtered air [19]. Additionally, diesel exhaust inhalation impaired endogenous fibrinolytic capacity in these patients with reduced endothelial release of tissue plasminogen activator (t-PA) six hours following exposure. The time course of these adverse vascular and prothrombotic effects is consistent with the observation of an immediate and delayed association between traffic exposure and the onset of acute myocardial infarction [20].

Diesel exhaust particles (DEP) generated under different engine running conditions vary in size and chemical composition. It is therefore of interest to elucidate the associations between physicochemical properties of the DEP and health effects caused by exposure to DE generated under various engine running conditions. In general, the mass of emitted DEP is often dominated by carbonaceous agglomerated soot particles, typically 50-300 nm, while the number concentration is dominated by smaller particles, below 50 nm, mainly composed of volatile organic material and sulphur compounds. Adsorbed organic compounds, as well as small amounts of sulphate, nitrate, metals and other trace elements are generally also associated with the diesel exhaust. Further, a fraction of fuel and lube oil generally escape oxidation in the engine and are emitted as exhaust PM, consisting of different organic compounds including polycyclic aromatic hydrocarbon (PAH) compounds [21,22].

People living in urban areas are mainly exposed to particulate emissions generated during acceleration and deceleration conditions rather than from idling engines. We have therefore designed a human exposure system using the standardized European Transient Cycle (ETC) urban sequence that better reflects the exposures generated in ambient urban settings. The aim of the present study was to determine whether inhalation of dilute diesel exhaust generated during the ETC urban sequence would affect vasomotor function and fibrinolytic capacity in healthy human subjects, in a similar or different way as following exposure to diesel exhaust generated under idling conditions.

## Methods

### Subjects

Eighteen healthy non-smoking males mean age 27 years (range 21-30 years) with normal lung function were included in the study, following a standardized clinical examination. All were free from symptoms of respiratory tract infections for at least 6 weeks prior to and during the study. Female subjects were not included due to the potential for cyclical hormones to affect vascular responses. The study was approved by the local ethics committee. All volunteers gave their written informed consent and the study was performed in accordance to the Declaration of Helsinki.

### Study design

The subjects were exposed to filtered air and diesel exhaust for one hour according to a randomized, double-blind, cross-over design with an interval of 42 (22-62) days between visits [15,23]. During each exposure, the subjects performed moderate exercise (average minute ventilation, 20 L/min/m^2 ^body surface) on a bicycle ergometer alternating with rest at 15-minute intervals.

Based on previous exposure studies exploring respiratory and cardiovascular effects of diesel exhaust [12,13,15,17], vascular assessments using venous forearm plethysmography were performed 6-hours after diesel and filtered air exposure. All subjects refrained from alcohol for 24-hours and from food and caffeine-containing beverages for at least 4-hours before each vascular study. They were not allowed to carry out strenuous exercise during the day of exposure. The studies were carried out in a quiet, temperature-controlled room maintained at 22°C to 24°C, with subjects in the supine position.

### Diesel exhaust exposure

**Figure 1:**
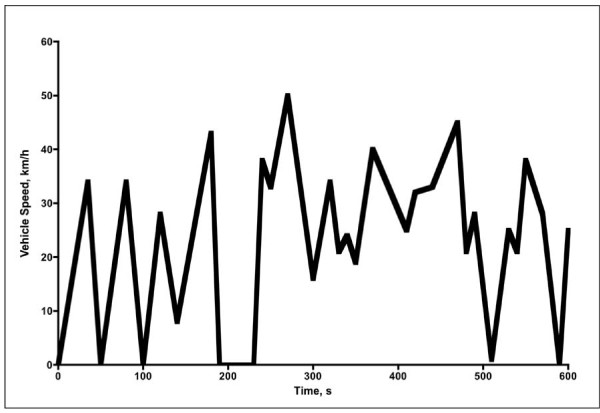
**The urban sequence of the European Transient Cycle applied in the present study**.

The exposures were performed in a specially built and validated human exposure chamber that has been described in detail elsewhere [23]. Diesel exhaust (DE) was generated by a diesel engine from 1991 (Volvo TD40 GJE, 4.0 L, four cylinders) connected to an engine dynamometer and running under control of a computer program according to the European Transient Cycle (ETC). The ETC is a well-established running condition for standardized tests of engines and vehicles and is designed to mimic real life running conditions for vehicles in an urban environment. As such, it is based on authentic recordings of accelerations and retardations, with variations in engine load as well as periods with constant speed. The running of the engine was controlled adjusting the actions of the injection system and an engine dynamometer that varied the load of the engine. The ETC test cycle has been introduced together with the European Stationary Cycle (ESC), for emission certification of heavy-duty diesel engines in Europe (Directive 1999/96/EC). The ETC comprises three different driving conditions, including urban, rural and motorway. In this study we used only the urban driving part, representing city driving with a maximum speed of 50 km/h, frequent starts, stops, and idling, i.e. transient engine running conditions (Figure 1).

The diesel fuel used was Statoil class 1; cetane number 54; aromatics, 4% volume; polycyclic aromatic hydrocarbons, < 0.02 vol-%; sulphur < 1ppm. The initial boiling point was 195°C and 95% volume boiling point was 280°C. More than 90% of the exhaust was shunted away and the remaining part was accumulated in an approximately 1 m^3 ^container in order to smooth the concentration variation during the transient running conditions. A partial flow of DE was continuously drawn from the container, diluted with filtered air and fed into the exposure chamber, giving a particle mass concentration of approximately 250 μg/m^3 ^in the exposure chamber.

The particle mass concentration (as PM_10_) of diesel exhaust particles (DEP) in the chamber during the exposures was measured gravimetrically employing standard glass fibre filter sampling as well as monitored with a Tapered Element Oscillating Microbalance (TEOM 1400) instrument (at 50°C). Measurements of NO_x_, NO and NO_2 _were performed with standard instruments using a chemiluminescence technique (ECO Physics CLD 700 AL Med, Switzerland). Total hydrocarbon in the exposure aerosol was measured using a flame ionization detection method (Hydrocarbon Analyzer, Model 3-300, JUM Engineering Co, Oakland, California, USA). Data on exposure characteristics are given in Table 1.

**Table 1:** Exposure characteristics of the present study during European Transient Cycle urban conditions compared to earlier studies [12,14] during idling engine conditions.

	ETC urban present study	Idling previous study
***Exposure campaigns***		
Diesel engine	Volvo TD40, 4.0 L, four cylinders	Volvo TD45, 4.5 L four cylinders
Diesel fuel	Diesel class I	Diesel class III
PM_10 _mass concentration (filter), μg/m^3^	254 ± 36	330 ± 12 ^(3)^
PM_10 _mass concentration (TEOM), μg/m^3^	228 ± 19	-
NO_x_, ppm	7.5 ± 0.3	2.8 ± <0.1 ^(3)^
NO_2_, ppm	0.9 ± 0.1	0.6 ± <0.1 ^(3)^
NO, ppm	6.6 ± 0.3	2.2 ± <0.1 ^(3)^
Total hydrocarbons (THC), ppm	1.2 ± 0.2	1.6 ± 0.2 ^(3)^
***Complementary measurements ^(1) ^***		
Particle number concentration, #/cm^3^	1.2 × 10^5^	9.5 × 10^5 ^^(4)^
Particle count median size (CMD_mobility_), nm	129	55 ^(4)^
Particle mass median size (MMD_aerodynamic_) nm	116	199 ^(4)^
Organic carbon fraction (OC/TC^(2)^), %	12	94.5 ^(4)^
Elemental carbon fraction (EC/TC^(2)^), %	88	5.5 ^(4)^
Total PAH concentration, μg/m^3^	0.96	3.5 ^(4)^
Semi-volatile PAH concentration, μg/m^3^	0.69	3.4 ^(4)^
PM-associated PAH concentration, μg/m^3^	0.27	0.16 ^(4)^

^(1) ^Particle number and PAH concentrations normalized to a PM mass concentration of 300 μg/m^3 ^to enable a comparison between ETC and idling conditions.

^(2) ^TC = Total carbon content in PM mass

^(3) ^Exposure protocol data from a previously reported study [14]

^(4) ^Un-published DEP characteristics data associated with the exposure conditions during the previously reported studies [12,14]

For the exposure campaign data, mean values are given mean ± std.dev.

**Figure 2:**
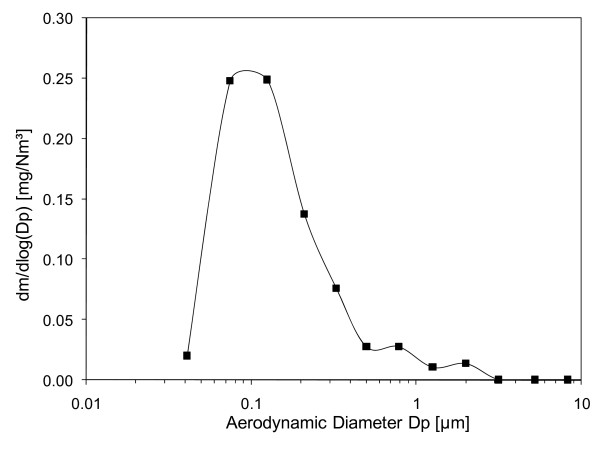
**Mass size distribution of diluted diesel exhaust particles in the exposure chamber during the urban running part of the European Trancient Cycle**. MMD_a _for the fine mode was 0.116 μm.

Extensive physicochemical characterisation of the DEP in the chamber was performed on a single occasion after the campaign, but under the same conditions as during the exposures. The aerodynamic particle mass size distribution in the range of 0.03-10 μm was determined using a 13 stage low-pressure cascade impactor (Dekati Ltd, Tampere, Finland). As seen in Figure 2, fine particles (< 1 μm) totally dominated the PM mass in the chamber and the fine mode aerodynamic mass median diameter (MMDa) was determined to be 116 nm (Table 1).

**Figure 3:**
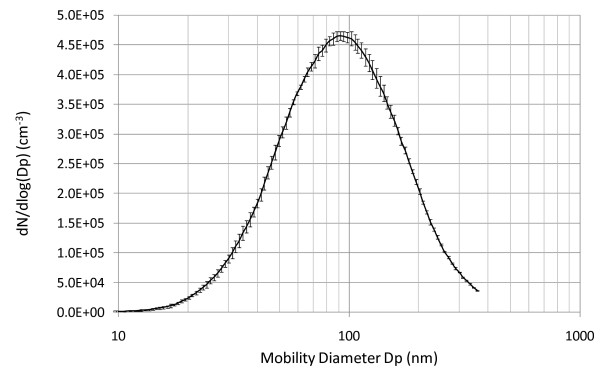
**Number size distribution of diluted diesel exhaust particles in the exposure chamber during the urban running part of the European Transient Cycle**. Standard deviations for 3 scans are shown as error bars. A log-normal fitting calculation showed that the size distribution comprised in principal only one single mode.

A scanning mobility particle sizer (SMPS) was used to classify the particles regarding their number, size distribution and concentration within 0.013 to 0.380 μm (equivalent mobility diameter). The system consisted of a differential mobility analyzer (DMA) (TSI model 3071A, TSI, St Paul, Minnesota, USA) and a condensation particle counter (CPC) (TSI model 3010, TSI, St Paul, Minnesota, USA). Relatively long scan times, i.e. 120 s up and 40 s down, were used to enable purging of the system between the scans. Aerosol flow was set to 1 L/min and sheath air flow to 7 L/min. The average total number concentration of fine particles in the chamber (normalized to a mass concentration of 300 μg/m^3^) and average count median (equivalent mobility) diameter (CMDm) are shown in Table 1 and Figure 3.

The DEP were characterized chemically regarding carbon fractionation (organic/elemental carbon), PAH and trace elements. The fraction of organic and elemental carbon of the PM in the chamber was determined by thermal-optical carbon analysis (Method NIOSH 5040) using quartz and teflon+quartz filters in parallel according to standard procedures [24]. "Elemental carbon" is in principle a measure of the soot fraction of samples and the result therefore implies that the mass of DEP during the present ETC conditions was dominated by soot particles (Table 1).

PAH were sampled by glass fiber filter sampling (diameter 47 mm) for the particulate fraction followed by a polyurethane foam (PUF) plug (diameter 75 mm × 50 mm) for the semi-volatile fraction. Forty-nine PAH compounds were analyzed by gas chromatography-mass spectrometry (GC-MS) for quantification in the particulate and semi-volatile fractions, respectively, according to procedures described in more detail elsewhere [25-27]. A total PAH concentration (normalized to a mass concentration of 300 μg/m^3^) of 0.96 μg/m^3 ^was determined in the chamber distributed as 0.69 μg/m^3 ^in the semi-volatile fraction and 0.27 μg/m^3 ^as particulate associated (Table 1). This implies that PAH constituted < 1% of the total DEP mass in the chamber. During the idling engine conditions, the PAHs contributing most to the atmosphere in the exposure chamber were phenanthrene and methyl derivatives of phenanthrene, equivalent to more than 80% of the PAHs determined. The corresponding value for the transient engine load (urban part of ETC) was more than 75%.

### Determination of oxidative properties of particles

DEP were collected using a high volume cascade sampler with a multi-stage round slit nozzle impactor, within the exposure chamber under both idling and transient (ETC urban part) engine conditions. Coarse and fine diesel particles were collected onto polyurethane foams by impaction [28] and extracted from the collection substrate with methanol using extensive vortexing and sonication, as described previously [29]. PM oxidative activity was assessed in a synthetic respiratory tract lining fluid (RTLF) on an equal mass basis (50 μg/mL). This involved establishing the capacity of fine (PM_0.1-2.5_) and coarse (PM_2.5-10_) samples to deplete ascorbate (AA), glutathione (GSH) and urate (UA), each at a starting concentration of 200 μM, over a 4-h incubation period (37°C). After the 4-h incubation period, samples were either acidified with metaphosphoric acid to a final concentration of 5% (w/v) for UA andAA measurement, or diluted into 100 mM phosphate buffer for the determination of glutathione (GSH). Methodological details for these antioxidant determinations have been described previously [30]. The final PM oxidative potentials (OP) were expressed as the percentage loss of either AA (OP^AA^) or GSH (OP^GSH^) over the 4-h incubation, relative to the particle free control, expressed per μg of extracted PM. The OP values associated with the coarse and fine fraction for each antioxidant were combined to derive an expression for PM_0.1-10_. Urate was not lost from the synthetic RTLF with any of the PM samples tested.

### Vascular studies

Forearm blood flow (FBF) was measured during unilateral brachial artery infusion of endothelial-dependent and -independent vasodilators using venous occlusion plethysmography with mercury-in-silicone elastomer strain gauges, as described previously [12]. Briefly, the brachial artery of one arm was cannulated with 27-standard wire gauge steel needle and 17 gauge venous cannulae were inserted in large veins in the antecubital fossa of both arms. Following a 30-minute baseline saline infusion, acetylcholine 5, 10, and 20 μg/min (endothelium-dependent vasodilator which does not release tissue plasminogen activator [t-PA]; Merck Biosciences); bradykinin at 100, 300, and 1,000 pmol/min (endothelium-dependent vasodilator which releases t-PA; Merck Biosciences); and sodium nitroprusside (SNP) 2, 4, and 8 μg/min (endothelium-independent vasodilator which does not release t-PA; David Bull Laboratories) were infused for 6 minutes at each dose with FBF measured for the last 3 minutes of each infusion. The infusions of the three vasodilators were given in a random order and separated by 20-minute saline infusions. Verapamil at 10, 30, and 100 μg/min (endothelium-and NO-independent vasodilator which does not release t-PA) was infused at the end of the study protocol due to its prolonged action. Blood pressure and heart rate were measured during the forearm study using a validated semi-automated oscillometric sphygmanometer.

Blood (10 mL) was withdrawn simultaneously from each arm at baseline and during infusion of each dose of bradykinin and collected into acidified buffered citrate (Stabilyte tubes, Biopool International) for tissue plasminogen activator (t-PA) assay. The samples were kept on ice until centrifuged at 2,000 *g *for 30 minutes at 4°C. Platelet-free plasma was decanted and stored at -80°C before assay. Plasma t-PA antigen concentrations were determined by ELISA (TintElize t-PA, Biopool EIA). Hematocrit was determined by capillary tube centrifugation at baseline and at the end of the study protocol.

### Markers of systemic inflammation

Peripheral blood samples were taken immediately before and 2 and 6 hours post-exposure and were analyzed for total and differential cell counts by an autoanalyzer in the local clinical biochemistry reference laboratory (Umeå University Hospital, Umeå, Sweden). Serum and plasma were prepared for measurement of interleukin-6 (IL-6), tumor necrosis factor-alpha (TNF-alpha), soluble P-selectin, soluble intracellular adhesion molecule-1 (ICAM-1) and CD40 ligand using commercially available ELISAs (Quantikine, R&D Systems). Serum C-reactive protein (CRP) was measured using an immunonephelometric assay (Behring BN II nephelometer).

### Data analysis and statistics

Plethysmographic data were analyzed as described previously [31]. The net release of t-PA antigen was defined as the product of the infused forearm plasma flow (based on the mean hematocrit and the infused FBF) and the concentration difference between the infused and non-infused arms as described previously [31]. Data were analysed by 2-way ANOVA with repeated measures or 2-tailed Student's *t*-tests, where appropriate, using GraphPad Prism (GraphPad Software, Version 4 for Macintosh) and SPSS (SPSS inc. Chicago, IL, USA, version 15). All data are expressed as mean ± SEM. Statistical significance was taken at p < 0.05.

## Results

### Exposure characteristics

Compared to the idling situation, exposure data from the ETC urban running condition revealed that the particles were larger but lower in number, despite similar mass concentration. During ETC, the fraction of organic carbon/elemental carbon was reduced, indicating higher soot content, and the total PAH concentration was reduced compared to the idling situation. Furthermore, NO_x _levels were increased under ETC, although the oxidative potential of the diesel particles was reduced (Table 1).

### Oxidative potential

Determination of oxidative potential (OP) of the PM samples demonstrated that DEP generated under the present ETC urban engine conditions were less oxidizing (PM_0.1-10 _OP^GSH ^= 0.15 ± 0.12%/μg, PM_0.1-10 _OP^AA ^= 0.25 ± 0.13%/μg) than DEP generated under presently studied idling engine conditions (PM_0.1-10 _OP^GSH ^= 0.43 ± 0.11%/μg, PM_0.1-10 _OP^AA ^= 0.43 ± 0.16%/μg, ETC *versus *idling engine). The oxidative properties observed from both these experimental tail-pipe emissions were minor compared with those observed in a previous study of ambient PM samples collected from roadside sites (OP^GSH ^= 0.60 ± 0.45 and OP^AA ^= 1.26 ± 0.37) [32].

### Vascular and fibrinolytic function

**Figure 4:**
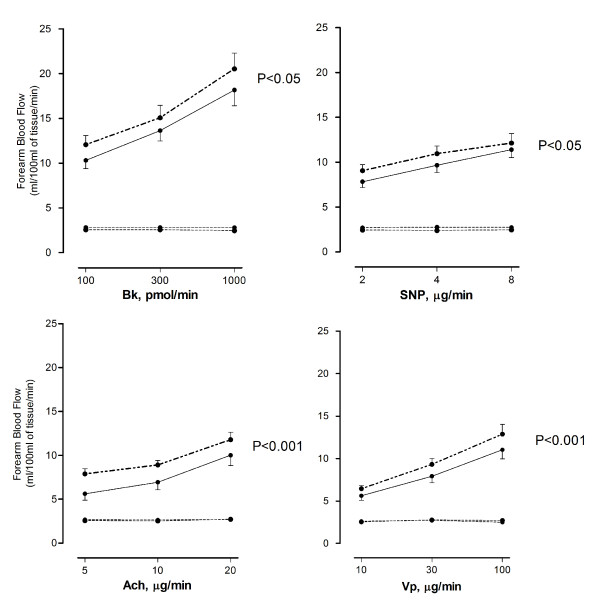
**Forearm blood flow measured six hours after diesel exhaust (solid line) and filtered air (dashed line) exposure during unilateral intra-brachial infusion of bradykinin (Bk), acetylcholine (Ach), sodium nitroprusside (SNP) and verapamil (Vp) in infused and non-infused (dotted lines) arms**. Effect of exposure compared with using 2-way ANOVA with repeated measures. Data plotted as mean ± SEM. P-values are given for diesel exhaust exposure vs. air.

**Figure 5:**
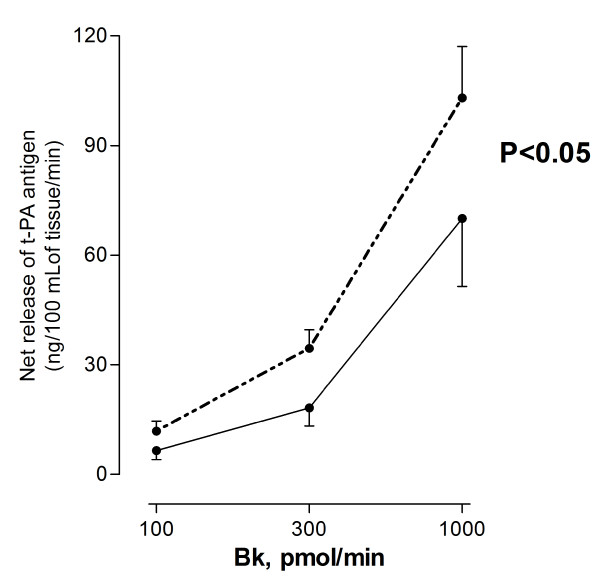
**Net release of t-PA antigen in the infused arm during unilateral intra-brachial infusion of bradykinin (Bk) after diesel (solid line) and air (dashed line) exposures**. Effect of exposure compared using 2-way ANOVA with repeated measures. Data plotted as mean ± SEM.

There were no differences in resting heart rate, blood pressure, or baseline FBF (P > 0.05 for all) after exposure to diesel exhaust or filtered air (Table 2). All infusions of vasoactive drugs increased blood flow in a dose dependent manner (P < 0.001). Diesel exhaust exposure significantly attenuated the increase in forearm blood flow during infusions of both endothelium-dependent and -independent vasodilators, as compared with filtered air exposure (P < 0.05 for bradykinin, P < 0.001 for acetylcholine, P < 0.05 for sodium nitroprusside and P < 0.001 for verapamil( Figure 4).The net release of t-PA following bradykinin infusion was reduced (P < 0.05) after exposure to diesel exhaust compared with filtered air (Figure 5).

**Table 2:** Baseline haemodynamic parameters after filtered air and ETC urban diesel exhaust exposures.

	Filtered air	Diesel exhaust
Heart rate, bpm	63 ± 1.7	63 ± 2.5
Systolic blood pressure, mmHg	142 ± 3.2	142 ± 2.7
Diastolic blood pressure, mmHg	68 ± 2.9	68 ± 1.8
FBF in infused arm, ml/100 ml tissue/min	3.1 ± 0.3	2.8 ± 0.3
FBF in non-infused arm, ml/100 ml tissue/min	2.5 ± 0.2	2.9 ± 0.3

Data expressed as mean ± SEM. P > 0.05 for all using paired Student's *t*-test

### Systemic inflammation

Peripheral blood leukocyte, neutrophil and platelet counts, as well as plasma concentrations of TNF-α, IL-6, soluble P-selectin, soluble ICAM-1, CD40L and CRP did not differ (P > 0.05 for all) between diesel exhaust and filtered air exposures (Table 3).

**Table 3:** Peripheral blood parameters after filtered air and diesel exhaust exposures.

	Before air exposure	6 hours after air exposure	Before diesel exposure	6 hours after diesel exposure
Leukocytes (× 10^9 ^cells/L)	5.6 ± 0.3	5.9 ± 0.3	6.0 ± 0.3	5.5 ± 0.2

Neutrophils (× 10^9 ^cells/L)	2.4 ± 0.2	3.6 ± 0.3	2.6 ± 0.2	3.1 ± 0.2

Platelets (× 10^9 ^cells/L)	226 ± 8	214 ± 8	229 ± 10	214 ± 8

TNF-α (pg/ml)	0.92 ± 0.21	0.89 ± 0.17	1.03 ± 0.17	0.80 ± 0.17

IL-6 (pg/ml)	0.68 ± 0.22	2.58 ± 0.82	0.60 ± 0.14	2.17 ± 0.76

CRP (mg/l)	1.32 ± 0.52	1.30 ± 0.46	2.03 ± 0.56	1.85 ± 0.56

CD40L (pg/ml)	95.4 ± 9.2	88.8 ± 10.8	98.4 ± 11.1	85.6 ± 11.1

P-Selectin (ng/ml)	52.0 ± 5.7	58.4 ± 7.1	57.4 ± 6.7	54.1 ± 7.4

ICAM-1 (ng/ml)	260.0 ± 13.9	273.1 ± 24.4	267.8 ± 14.2	252.8 ± 17.4

Data expressed as mean ± SEM. P > 0.05 for all using 2-way ANOVA with repeated measures.

## Discussion

We demonstrate that short-term exposure to diesel exhaust generated under urban transient engine running conditions, mimicking a 'real-world' urban environment, impairs vascular vasomotor function and endogenous fibrinolytic function in human subjects. These vascular effects are consistent with those reported in studies employing a similar study protocol, but using an idling engine. This suggests that different diesel engine running conditions induce similar adverse vascular responses.

**Figure 6:**
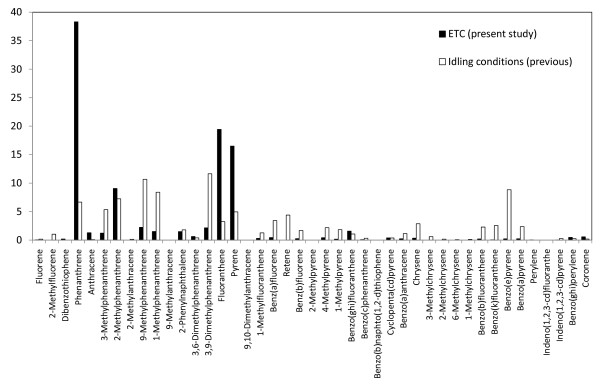
**Relative distribution (in % of total PAH) for specific particulate associated PAH compounds for the present ETC running conditions in comparison with idling conditions used in previous studies **[12,14].

**Figure 7:**
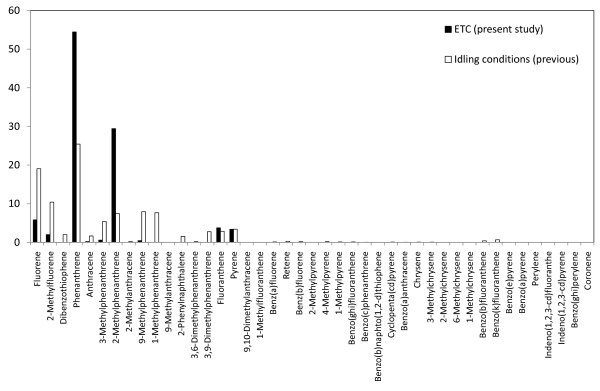
**Relative distribution (in % of total PAH) for specific semi-volatile PAH compounds for the present ETC running conditions in comparison with idling conditions used in previous studies **[12,14].

The ETC urban running condition was selected for this study as it is based on actual recordings from vehicles, and therefore provides a highly relevant model of ambient air pollution exposures in urban settings. Whilst the vascular responses were similar after ETC urban running conditions and idling, it was found that the chemical and physical properties differed between exposure situations. The particles generated under the transient running setting were slightly larger (according to the mobility diameter, related to the number concentration) and had a higher elemental carbon to organic carbon ratio, i.e. more soot and less organics, than those generated from the idling diesel engine. The PAH profiles in the present study were also in good agreement with previous diesel PAH exhaust studies [26,33]. Furthermore, when comparing the PAH profiles from the idling and the transient running conditions, they appeared similar, but with the former having more than 4 times greater concentrations of sum PAHs (ng/m^3^) in the exposure chamber, mainly related to higher idling gaseous PAH concentrations. In contrast, PM-associated PAH concentrations were similar, but differed in profile, with higher concentrations of phenanthrene, fluoranthene and pyrene under transient load and speed conditions (Figure 6). PM-associated PAHs present under idling conditions were distributed as a higher number of compounds, including heavier PAH (4-rings) species (Figure 7). This can be explained by the fact that the engine's combustion temperature is at its lowest value when operating at idling mode. The relatively larger hydrocarbon emissions at idling reflect unburned fuel constituents, indicating that the major part of the PAHs in the exhaust originates from PAHs originally in the diesel fuel. It has previously been shown that PAHs in the diesel fuel represent a significant source for exhaust PAHs, besides the pyrosynthesis of exhaust PAH [33,34].

The present study demonstrates that inhalation of diesel exhaust generated under urban ETC running conditions, causes a reduction in endothelium-dependent and endothelium-independent vasodilatation, together with an impairment of t-PA release from the vascular endothelium. Perturbations of vascular homeostasis are thought to be important in the triggering of acute myocardial infarction and stroke [8,35]. The vascular assessment in the present study was identical to that used in previously reported studies employing diesel exhaust generated under idling conditions [12,13] and was intended to enable comparisons between studies. The mass concentration of diesel exhaust particles within the exposure chamber differed slightly between the ETC and idling exposures (254 versus 330 μg/m^3^), as did the particle properties. Irrespective of a slightly lower PM mass, difference in particle size and composition, as well as a lower concentration of PAHs in the present study, we demonstrated similar adverse vascular responses following exposure to diesel exhaust generated by an engine under the ETC running conditions compared to the idling situation.

Interestingly, the present investigation demonstrated that vasodilatation in response to intra-arterial verapamil was impaired following diesel exhaust generated under transient running conditions. This suggests that the vascular smooth muscle also demonstrates a calcium flux-dependent vasomotor function disturbance, which is not endothelium-dependent. This has not previously been shown in studies with idling diesel exhaust that has a higher content of gaseous (semi volatile) PAH and contains a lower fraction of soot particles than the exhaust generated under ETC urban conditions. It can thus be speculated that the higher diesel-related soot content and its associated (adsorbed) organic material in exhaust generated under transient running conditions may cause additional vascular smooth muscle effects, but this issue will need further investigation.

The oxidative capacity of diesel exhaust particles generated under idling conditions has previously been confirmed by analysis of bronchoalveolar lavage and bronchial mucosal biopsies sampled from human subjects, as well as *in vitro *studies [30]. Of note, the oxidative potential of diesel exhaust particles generated in the present study was less pronounced compared to the previous idling situation. Whilst certain differences in particle size, organic carbon/elemental carbon-ratio, PAH-content and oxidative potential exist between the two exposure situations, the cardiovascular effects were generally similar following ETC running conditions although the verapamil-induced vasodilatation was at variance to our previous studies. One could speculate that other exhaust components (particle associated or gaseous) may be the causal link between the exposure and the different adverse vascular effects. In this context, Xia *et al *suggest that organic substances such as quinones may be important in mediating some of the cardiovascular effects of diesel exhaust [36]. Quinones and semi-quinones generated during combustion or cell metabolism have strong oxidative capacity and may interact with mitochondria and affect membrane potential and cell breathing [36,37]. This could be mechanistically linked to diesel exhaust-induced vascular events, as well as the increase in ST-segment depression, identified in patients with stable coronary disease [19]. Accordingly, it is important to further elucidate the role of quinones in mediating the adverse effects of combustion-related air pollution.

There was a difference in NO_x _concentration and the ratio NO/NO_2 _between the present and previous study employing diesel exhaust exposure at idling. The published literature strongly suggests that the vascular effects present in this and previous experimental exposure studies are due to the presence of combustion-derived particles in diesel exhaust [8]. It has also been recently shown that exposure to NO_2 _does not affect vascular function [38], which further implies the importance of a particulate-related source for the vascular effects following exposure to diesel exhaust. However, further studies employing particle concentrator technologies or filtration systems are necessary to clarify the specific role of the particulate and gaseous components, other than NO_x_, in diesel exhaust.

Diesel exhaust induces a complex response in the human lungs, including oxidative stress, activation of redox-sensitive transcription factors, inflammatory cell influx, cell activation and secretion of a range of pro-inflammatory components [15,17,32,39]. It has been suggested that this pulmonary inflammation would "spill-over" to the circulation, thereby causing systemic effects. Systemic inflammation has indeed been implicated as an important component in atherosclerosis, vasomotor dysfunction and coronary events [30]. However, in the present investigation we were unable to demonstrate any increased systemic inflammation as reflected by CRP, CD40L, sP-selectin, sICAM-1, IL-6 and TNF-alpha levels in blood at 6 hours post-exposure. This is in line with our previous experiences from diesel exhaust exposures in humans that the presence of major inflammatory events reflected in the blood is not present during the early stages after exposure, but in the later stage of 18-24 hours coinciding with the peak inflammatory bronchoalveolar response [13,17]. This suggests that the diesel exhaust-induced vascular responses described here and in previous diesel exhaust studies using an idling engine are not related to a systemic inflammatory response.

It has been implied that inhaled diesel exhaust particles might directly elicit adverse effects outside the lungs, as inhaled particles are believed to have the potential to translocate from the alveoli to the vascular system and subsequently cause effects in the vasculature, heart and other organs. However, in humans, particle translocation has not yet been consistently proven [40-42]. Small numbers of diesel exhaust particles with their complex highly reactive surfaces, or soluble components from these particles, may well reach the circulation, affecting the endothelium in the pulmonary and peripheral vasculature.

Apart from the running conditions, the exposure protocol was the same as in previous studies and a similar particle mass concentration was intended to enable comparisons. It is recognised that a direct comparison would benefit from having the different engines and running modes included within the same study. This was not possible due to practical issues, and would have demanded even more extensive resources with dual engines mounted, as well as multiple exposures and invasive vascular measurements in the same individuals.

It is concluded that exposure to diesel exhaust generated during urban transient running conditions, a highly relevant model of urban air pollution, impairs vasomotor function and endogenous fibrinolysis. These findings are consistent with previous studies employing an idling engine and indicate that adverse vascular effects of diesel exhaust inhalation occur over different running conditions with varying gaseous and particle composition and concentrations as well as physicochemical particle properties. Of note, exposure to diesel exhaust under urban running conditions was also associated with a disturbance of calcium channel-dependent vasomotor function, not previously demonstrated. This may suggest that certain cardiovascular endpoints following exposure to diesel exhaust are related to exhaust properties in common between the two studied engine running conditions, whereas the novel calcium flux-related effect seems to be associated with exhaust properties more specific for transient load and speed (ETC) conditions, for example a higher content of diesel soot particles and their adsorbed organic compounds.

## List of abbreviations

AA: Ascorbic Acid; Ach: Acetylcholine; Bk: Bradykinin; Bpm: Beats per minute; DE: Diesel exhaust; DEP: Diesel exhaust particles; EC: Elemental carbon; ETC: European transient cycle; FBF: Forearm blood flow; GSH: Reduced glutathione; OP: Oxidative potential; NO: Nitric oxide; NO_x: _Oxides of nitrogen; NO_2: _Nitrogen dioxide; OC: Organic carbon; PAH: Polycyclic Aromatic Hydrocarbons; PM: Particulate matter; SNP: Sodium nitroprusside; TC: Total carbon; t-PA: Tissue plasminogen activator; Vp: Verapamil.

## Competing interests

The authors declare that they have no competing interests.

## Authors' contributions

SB, AB, NM, KD, TS and DN coordinated and were responsible for the planning of the study. NM, AL and SB were responsible for the diesel exhaust exposures. CB and JB were responsible for the exposure characteristics. IM carried out and interpreted data on oxidative potential and made input on manuscript. RW performed the PAH characteristics. SB and NM analysed data and performed statistical analysis. The manuscript was written by SB, NM and AB and then read, corrected and approved by all authors.
